# 601. Clinical Presentation and Pregnancy Outcomes of PCR-Proven Rickettsial Infections in Pregnant Women

**DOI:** 10.1093/ofid/ofad500.668

**Published:** 2023-11-27

**Authors:** Yael Yagel, Tal schlaeffer Yosef, Yonat Shemer-Avni, Ayelet Keren-Naos, Lior Nesher

**Affiliations:** Soroka Medical Center, Beer Sheba, HaDarom, Israel; Soroka University Medical Center, Ben-Gurion University of the Negev, Beer Sheba, HaDarom, Israel; Soroka University Medical Center, Clinical Virology Laboratory, Beer Sheva, HaDarom, Israel; Soroka University Medical Center, Clinical Virology Laboratory, Beer Sheva, HaDarom, Israel; Soroka Medical Center, Beer Sheba, HaDarom, Israel

## Abstract

**Background:**

Rickettsial infections are endemic zoonoses found worldwide. Spotted fever (*R. conorii)* and murine typhus (*R. typhi)* cause a spectrum of diseases from mild to multi-organ failure and death. Diagnosis and treatment of rickettsial infections in pregnancy are challenging, and little is known about pregnancy outcomes. We describe a cohort of PCR-proven rickettsial infections in pregnant women in Israel.

**Methods:**

Whole blood samples were taken from pregnant women with acute febrile illness. Samples were subjected to DNA extraction and a general set of primers and probe was used to identify Rickettsia spp. by RT-PCR.

**Results:**

In 2020, 12 pregnant women were diagnosed by PCR with rickettsial disease. Upon presentation, the mean age was 26 (18-39) years, and the mean gestational age was 31 (22-40) weeks. The number of days until diagnosis was established was 5 (2-10). The most common symptom other than fever was headache (11/12), only half presented with a rash. Most women developed elevated liver enzymes (11/12), and 2/12 had thrombocytopenia. Interestingly, 7/12 presented with respiratory symptoms, and 2/12 had myocardial involvement manifested as either elevated myocardial enzymes or ventricular dysfunction on echocardiogram. All women received Azithromycin as a first-line anti-rickettsial antibiotic, while 5 women were switched to Doxycycline due to disease severity. Defervescence within 24 hours after a single antibiotic dose was seen in 8/12 of women. Only 1/12 were delivered preterm, and the mean birth weight was 3000g. Serologic assays were available for 11/12 women, confirming murine typhi in 5/12 cases, and unidentified rickettsial infection in 3/12. Species identification wasn't available using PCR alone.

**Conclusion:**

Molecular assays are a valuable tool for real-time diagnosis of rickettsial infections in pregnant women, allowing for a rapid targeted antibiotic treatment with good clinical outcomes for both mother and neonate. Unusual rates of respiratory and myocardial involvement were observed in our cohort, suggesting a possible specific pregnancy-related complication.

**Disclosures:**

**All Authors**: No reported disclosures

